# l-Lysine supplementation affects dietary protein quality and growth and serum amino acid concentrations in rats

**DOI:** 10.1038/s41598-023-47321-3

**Published:** 2023-11-15

**Authors:** Chao-Wu Xiao, Amy Hendry, Laura Kenney, Jesse Bertinato

**Affiliations:** 1 Nutrition Research Division, Food Directorate, Health Products and Food Branch, Health Canada, Banting Research Centre, Ottawa, ON K1A 0K9 Canada; 2 Food and Nutrition Science Program, Department of Chemistry, Carleton University, Ottawa, ON K1S 5B6 Canada; 3 Department of Biochemistry, Microbiology and Immunology, University of Ottawa, Ottawa, ON Canada

**Keywords:** Biochemistry, Physiology

## Abstract

Single amino acid (AA) supplementations in foods are increasing, however their potential nutritional and physiological impacts are not fully understood. This study examined the effects of l-lysine (Lys) supplementation on protein quality of diets, serum AA concentrations and associations between the ratio of supplemental Lys to dietary protein (X) with body weight gain (BWG) in Sprague–Dawley male rats. Rats were fed one of 10 diets containing either 7% or 20% casein and supplemented with 0% (Control), 1.5%, 3%, 6% Lys or 6% Lys + 3% l-arginine (Arg) (8 rats/diet group) for 1 week. Lys supplementation reduced the protein quality of the casein-based diets (p < 0.01). BWG was reduced by supplemental Lys when X > 0.18. Free Lys supplementation dose-dependently increased serum Lys levels (p < 0.01), while increased protein-bound Lys (1.4% vs 0.52%) had little effect on serum Lys (p > 0.05). In the 7% casein diets, ≥ 1.5% supplemental Lys reduced serum alanine, asparagine, glycine, isoleucine, leucine, serine, tyrosine, valine, carnitine, ornithine, and increased urea. Supplementation of ≥ 3% Lys additionally reduced tryptophan and increased histidine, methionine and α-aminoadipic acid (α-AAA) compared to the Control (p < 0.05). In the 20% casein diets, addition of ≥ 1.5% Lys reduced serum asparagine and threonine, and ≥ 3% Lys reduced leucine, proline, tryptophan, valine, and ornithine, and 6% Lys reduced carnitine, and increased histidine, methionine, and α-AAA. Overall, this study showed that free Lys supplementation in a Lys-sufficient diet reduced the protein quality of the diets and modified the serum concentrations of many amino acids. Excess free Lys intake adversely affected growth and utilization of nutrients due to AA imbalance or antagonism. Overall lower protein intake increases susceptibility to the adverse effects of Lys supplementation.

## Introduction

l-Lysine (Lys) is an essential amino acid (EAA) in mammals, and must be obtained from foods or dietary supplements since it cannot be synthesized in the body^1^. Lys is usually low in cereal-based food products^2^, but rich in animal food sources such as lean beef, chicken, pork, and shellfish^1^. The Lys requirement in healthy adults is 30 mg/kg BW/day^3^, and Lys intake from Western diets is about 40–180 mg/kg BW/day^4^. The average Lys intake of Americans is 75.3 mg/kg BW/day^5^, which is over two times the daily requirement. Besides its nutritional roles, Lys has garnered popularity as a supplement in foods and drinks for its purported functional properties.

Lys has been used as a common workout supplement because of its potential roles in energy production and stimulation of muscle growth. Lys is a precursor of carnitine that is involved in mitochondrial β-oxidation to convert fatty acids into energy in mammals^6^. Moreover, Lys was shown to reduce protein degradation^7^ and stimulate protein synthesis^8,9^. Lys stimulated growth hormone release when infused intravenously or administered orally^10,11^. It is believed that supplementation with Lys before strength training workouts could increase exercise-induced growth hormone release, improving athletic performance by increasing muscle mass and strength^12,13^.

Lys supplement may prevent the recurrence of certain viral infections such as herpes simplex, shingles, and human papilloma virus, and improve the associated symptoms^14–16^. Lys and l-arginine (Arg) share the same Lys/Arg transporters and therefore compete for uptake into cells. Excess Lys can decrease intracellular Arg levels. Replication of certain viruses requires higher Arg and lower Lys. Increasing cellular Lys concentrations disrupts the metabolic balance between Lys and Arg, and inhibits viral replication^17^.

Addition of Lys to foods containing high starch and low protein such as fabricated snack products reduces formation of acrylamide via Maillard reaction when processed at high temperature^18^. Asparagine content in these foods was positively associated with acrylamide formation in a dose-dependent manner^19^. When Lys was added prior to frying, the formation of acrylamide was eliminated^20,21^. Kim et al. showed that pre-treatment of snack food products with 3% Lys prior to frying reduced acrylamide formation by 80%^22^. It was postulated that the ε-NH_2_ group of Lys reacts with acrylamide to form a Lys-acrylamide adduct in a 2:1 ratio, thereby eliminating the formation of acrylamide^21,23^. Lys is currently allowed to be added in fabricated snack foods up to a level of 1.2% to reduce the formation of acrylamide.

Nutritional quality of a protein in diets is assessed by comparing the ratios of its EAA content to a reference protein or their requirements in humans or animals and corrected with protein digestibility of the diet^24^. The lowest EAA ratio in a protein is defined as the amino acid score. The EAA that has the lowest ratio in a diet is the first limiting EAA, and it limits the utilization of other EAA and determines the quality of the protein in the diet. Excess Lys supplementation could alter the ratios of the EAA and affect protein quality of the diets, and result in amino acid imbalance thereby adversely affecting physiological functions^25–27^. Our previous studies showed that the effects of Lys supplementation were modified by protein content in diets. The rats fed lower dietary protein were less tolerant to Lys supplementation and showed adverse effects such as decreased food intake and retarded growth^28,29^. However, the mechanism(s) involved, and the tolerable upper levels of Lys supplementation remain to be determined.

The nutritional safety and health effects of supplemental Lys intake are not fully understood. In this study, using rats as a model, we examined, (a) the effects of Lys supplementation in the context of a low or normal protein diet on protein digestibility and quality of the diets, (b) the effects of dietary protein-bound vs. free Lys on serum free amino acids and metabolites, (c) the association of the ratios of supplemental Lys to protein content in diets (X) with protein quality and body weight gain (BWG), and (d) the maximum level of supplemental Lys in terms of its ratio to dietary protein content that has no significant effects on BWG.

## Results

### EAA content and limiting EAA scores in diets

Dietary EAA content and scores are shown in Table 1. l-methionine (Met) + l-cysteine (Cys) were the limiting EAA in both the 7% and 20% casein diets and their amino acid scores were 0.71 and 0.69, respectively. Lys supplementation dose-dependently reduced the limiting EAA (Met + Cys) scores in the diets, and the extent of reduction by the same amount of supplemental Lys was higher in the 7% casein than in the 20% casein diet (Table 1). Supplementation with 1.5%, 3%, 6% Lys or 6% Lys + 3% Arg in 7% casein diets reduced the limiting EAA scores by 18%, 32%, 56% and 68% compared to the Control, respectively. The corresponding values for diets with 20% casein were 9%, 16%, 33% and 43% (Table 1).

**Table 1:** Dietary essential amino acid (EAA) content and ratios.

mg/g protein	Rat EAA req*	7% casein					20% casein				
		0	1.5% Lys	3% Lys	6% Lys	6%Lys + 3%Arg	0	1.5% Lys	3% Lys	6% Lys	6%Lys + 3%Arg
Arginine	29	32.2	25.8	22.5	15.7	151.7	34.3	31.3	29.9	24.4	107.7
Histidine	19	29.0	23.3	20.4	14.1	10.1	27.6	25.3	23.9	19.7	16.9
Isoleucine	41	51.5	41.8	36.5	25.1	18.0	49.5	45.1	42.4	35.0	29.9
Leucine	71	98.2	78.6	66.5	47.1	34.0	92.3	84.3	79.7	65.7	55.8
Lysine	61	83.7	199.0	289.7	371.0	255.2	78.8	126.5	170.3	225.0	191.1
Met + Cys	65	46.7	38.1	31.1	20.4	14.9	45.1	41.2	37.8	29.9	25.6
Phe + Tyr	68	80.5	65.1	56.9	39.2	28.6	95.7	85.8	82.4	68.5	58.1
Threonine	41	43.5	34.4	29.0	20.4	14.3	46.2	37.2	35.0	28.7	24.3
Tryptophan	13	14.5	9.8	9.7	6.3	5.3	15.2	11.4	11.0	10.6	8.1
Valine	49	66.0	52.8	45.1	31.4	22.8	62.5	57.5	53.9	44.5	37.6
EAA scores											
Arginine		1.11	0.89	0.78	0.54	5.23	1.18	1.08	1.03	0.84	3.71
Histidine		1.55	1.25	1.09	0.76	0.54	1.48	1.36	1.28	1.05	0.90
Isoleucine		1.25	1.01	0.88	0.61	0.44	1.20	1.09	1.02	0.85	0.72
Leucine		1.38	1.10	0.93	0.66	0.48	1.29	1.18	1.12	0.92	0.78
Lysine		1.37	3.24	4.72	6.05	4.16	1.29	2.06	2.78	3.67	3.12
Met + Cys		0.71	0.58 (− 18%)**	0.48 (− 32%)	0.31 (− 56%)	0.23 (− 68%)	0.69	0.63 (− 9%)	0.58 (− 16%)	0.46 (− 33%)	0.39 (− 43%)
Phe + Tyr		1.18	0.96	0.84	0.58	0.42	1.41	1.26	1.21	1.01	0.85
Threonine		1.05	0.83	0.70	0.49	0.35	1.12	0.90	0.85	0.69	0.59
Tryptophan		1.11	0.76	0.74	0.48	0.41	1.17	0.88	0.85	0.82	0.62
Valine		1.34	1.07	0.91	0.64	0.46	1.27	1.17	1.09	0.90	0.76

*Rat EAA requirement^30^.

**Percentage reduction of the limiting EAA scores compared to respective Control.

### Fecal true protein digestibility (TPD) and PDCAAS

**Figure 1:**
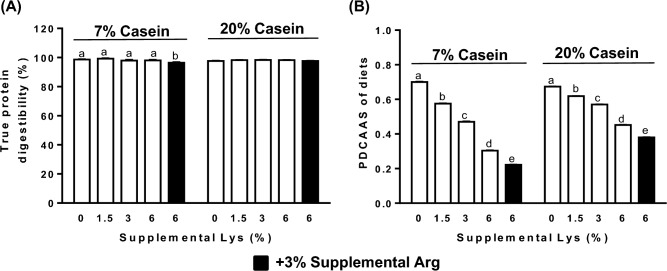
Fecal true protein digestibility (**A**), and protein digestibility-corrected amino acid scores (PDCAAS) (**B**) of the diets containing either 7% or 20% casein and supplemented with 0 (Control), 1.5%, 3%, 6% Lys (open bar) or 6% Lys + 3% Arg (filled bar). Values are means ± SEM, n = 8. Means with different letters in the same dietary casein content (7% or 20%) differ, p < 0.05.

Lys supplementation in both the 7% and 20% casein diets did not affect the fecal protein digestibility in the rats (p > 0.05). However, addition of 3% Arg along with 6% Lys in the 7% casein diet significantly lowered the fecal protein digestibility (p < 0.05, Fig. 1A). PDCAAS of the diets containing either 7% or 20% casein were dose-dependently lowered by increasing levels of supplemental Lys compared to their respective Controls (p < 0.05, Fig. 1B). Addition of 3% Arg in the 7% or 20% casein diets further lowered their PDCAAS over that of the 6% Lys alone (p < 0.05).

### Associations of ratios of supplemental Lys to dietary protein content with protein quality and BWG

The crude protein content and the ratios of supplemental Lys to the crude protein in diets (X) increased with Lys supplementation compared to the Controls. Addition of 3% Arg further increased crude protein content in the diets (Supplementary Table 2).

**Figure 2:**
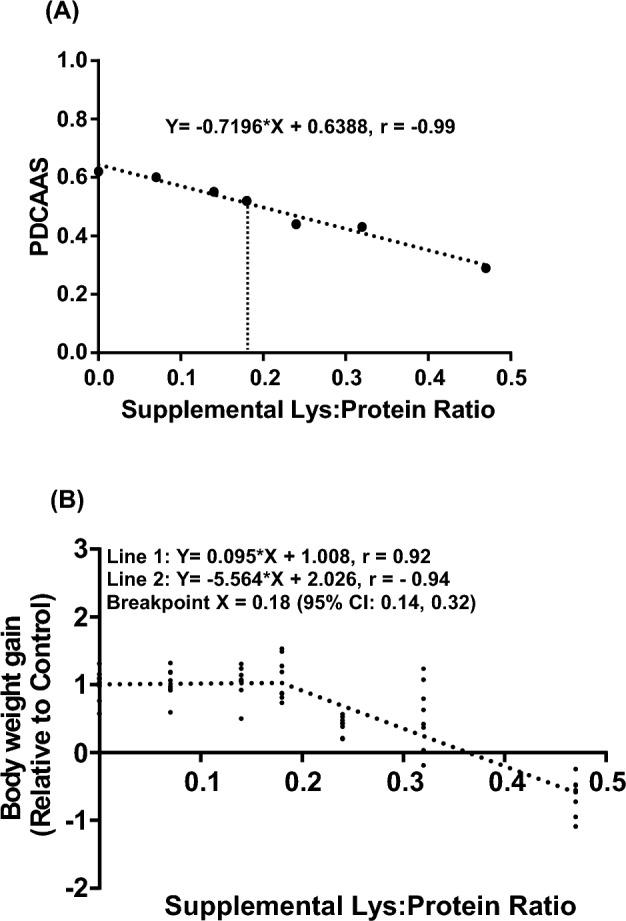
Correlations of the ratios of supplemental Lys to crude protein content (X) in diets with protein digestibility-corrected amino acid scores (PDCAAS) (**A**) and body weight gain (**B**) in the rats fed diets containing either 7% or 20% casein supplemented with 0, 1.5%, 3%, 6% Lys or 6%Lys + 3% Arg for 1 week. Values are means ± SEM, n = 8.

The protein quality of the diets, as measured by PDCAAS, were inversely correlated with X (Y = -0.7196*X + 0.6388, r = − 0.99, p < 0.0001, Fig. 2A). X showed a two-phase linear regression with BWG (Line 1: Y = 0.095*X + 1.008, r = 0.92; Line 2: Y = − 5.564*X + 2.026, r = − 0.94, p < 0.01, Fig. 2B). The inflection point for the two-phase linear regression with BWG occurred at 0.18, with 95% confidence intervals of (0.14, 0.32). When X > 0.18, the BWG was markedly reduced. Based on the regression equation Y = − 0.7196*X + 0.6388, the corresponding PDCAAS in the diets that would not result in a significant reduction in BWG was estimated to be 0.51.

### Serum Lys and Arg levels

**Figure 3:**
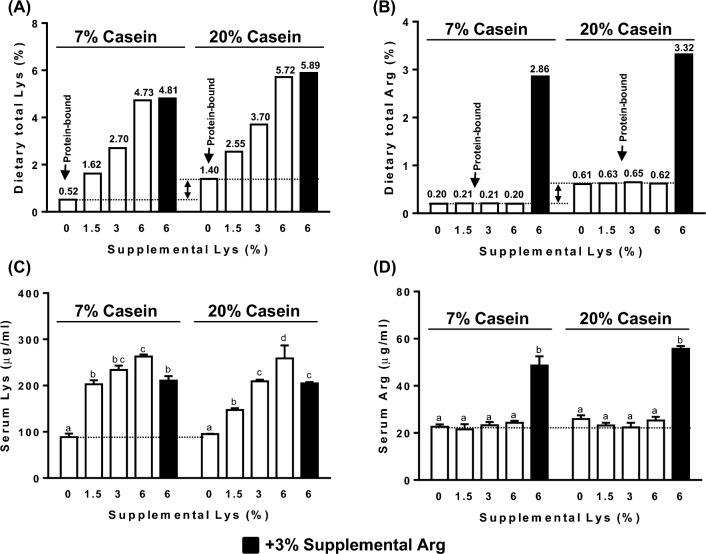
Protein-bound and supplemental free lysine (Lys) (**A**) and arginine (Arg) (**B**) content in the diets containing 7% or 20% casein supplemented with 0, 1.5%, 3%, 6% Lys (open bar) or 6% Lys + 3% Arg (filled bar). Serum free Lys (**C**), Arg (**D**) concentrations in the rats fed diets containing 7% or 20% casein supplemented with 0, 1.5%, 3%, 6% Lys (open bars) or 6% Lys + 3% Arg (filled bars) for 1 week. Values are mean ± SEM, n = 8. Means with different letters in the same dietary casein content (7% or 20%) differ, p < 0.05.

Lys supplementation in the diets dose-dependently increased serum levels of Lys compared to the Controls (p < 0.01, Fig. 3C). Addition of 3% Arg to the diets containing 7% or 20% casein supplemented with 6% Lys significantly increased serum Arg (p < 0.01, Fig. 3D), and lowered serum Lys to levels similar to those in the rats fed 1.5% or 3% supplemental Lys, respectively (Fig. 3C). Serum Lys (Fig. 3C) and Arg (Fig. 3D) concentrations in the rats fed different levels of protein-bound Lys (i.e. 0.52% in 7% casein diet vs 1.40% in 20% casein diet, Fig. 3A) or Arg (0.20% in 7% casein diet vs 0.61% in 20% casein diet, Fig. 3B) did not differ. The same levels of Lys supplementations at 1.5% or 3% resulted in higher retention of serum Lys in rats fed the 7% casein diet compared with rats fed the 20% casein diet (Fig. 3C).

### Other serum amino acids and metabolites

**Figure 4:**
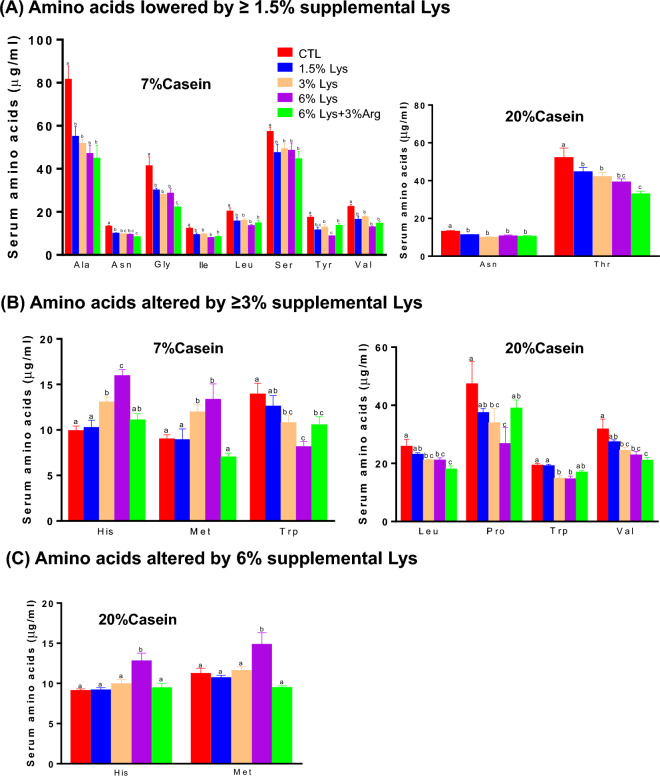
Serum concentrations of amino acids significantly altered by ≥ 1.5% (**A**), ≥ 3% (**B**) or 6% (**C**) supplemental Lys in male Sprague–Dawley rats fed diets containing either 7% or 20% casein and supplemented with 0, 1.5%, 3%, 6% Lys or 6% Lys + 3% Arg for 1 week. Values are mean ± SEM, n = 8. Means with different letters differ, p < 0.05.

**Figure 5:**
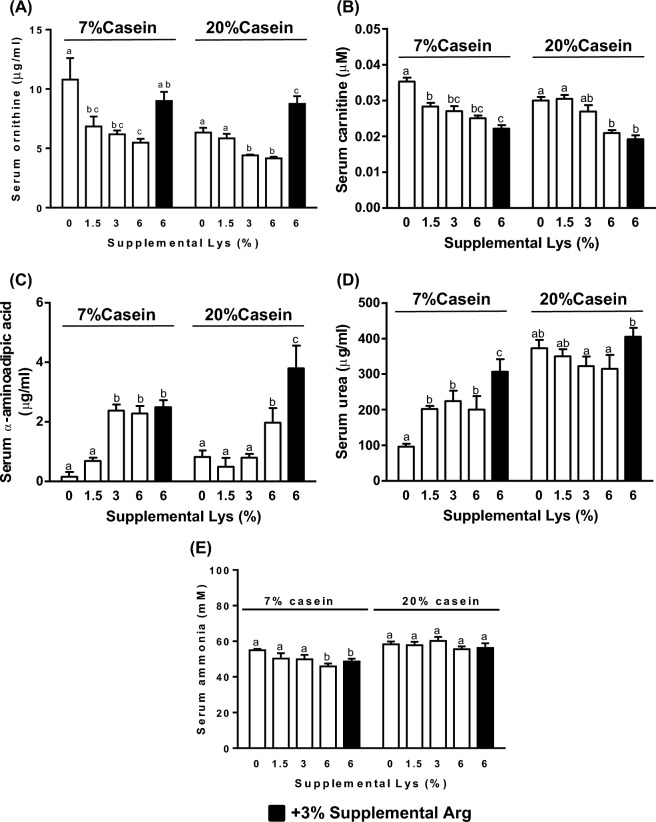
Serum ornithine (**A**), carnitine (**B**), α-aminoadipic acid (**C**), urea (**D**) and ammonia (**E**) concentrations in the male Sprague–Dawley rats fed diets containing either 7% or 20% casein and supplemented with 0, 1.5%, 3%, 6%Lys (open bars) or 6% Lys + 3% Arg (filled bars) for 1 week. Values are means ± SEM, n = 8. Means with different letters in the same dietary casein content (7% or 20%) differ, p < 0.05.

The effects of supplemental Lys on serum Gly, Thr, Tyr, α-aminoadipic acid (α-AAA), carnitine, and urea levels were modified by dietary protein content (p < 0.05). In the 7% casein diets, supplementation with ≥ 1.5% Lys lowered serum Ala, Asn, Gly, Ile, Leu, Ser, Tyr, Val (Fig. 4A), carnitine (Fig. 5A), and ornithine (Fig. 5B), and increased urea (Fig. 5D), and ≥ 3% Lys further reduced Trp, and increased His, Met (Fig. 4B) and α -AAA (Fig. 5C) compared to the Controls (p < 0.01). Addition of 3% Arg along with 6% Lys increased serum Tyr (Fig. 4A), ornithine (Fig. 5A) and urea (Fig. 5D) levels (p < 0.05) and lowered His and Met concentrations (p < 0.01, Fig. 4B) compared to the 6% Lys alone. Serum ammonia levels were lower in rats fed the 7% casein diet with either 6% Lys or 6% Lys + 3% Arg supplementations compared with the Control (p < 0.05, Fig. 5E).

In the 20% casein diets, ≥ 1.5% supplemental Lys reduced serum Asn and Thr levels (Fig. 4A), and ≥ 3% Lys additionally lowered serum Leu, Pro, Trp, Val (Fig. 4B), and ornithine (Fig. 5A) compared to the Controls. Serum His and Met (Fig. 4C) and α-AAA (Fig. 5C) were increased, whereas carnitine was reduced (Fig. 5B) by 6% supplemental Lys (p < 0.01). Addition of 3% Arg with 6% Lys significantly reduced His and Met levels (Fig. 4C), while it increased ornithine, α-AAA and urea levels compared to the 6% Lys alone (Fig. 5A,C and D, p < 0.01).

## Discussion

This study assessed the effect of Lys supplementation above Lys intakes needed to meet requirements. The Lys ratios in comparison with requirements in rats in the 7% and 20% casein-based diets used in the present study were 1.37 and 1.29, indicating that Lys was sufficient to meet the requirement of growing rats^30^. The sulfur-containing amino acids, Met and Cys, had the lowest ratios compared to other EAA, and were the limiting amino acids, even though the 7% or 20% casein diets were supplemented with 0.105% or 0.3% l-cystine, respectively.

Supplementation with increasing amounts of Lys in the Lys-sufficient casein diets dose-dependently lowered the protein quality as evidenced by reduced limiting amino acid (Met + Cys) scores. The extent of reduction in the limiting EAA scores and protein quality by the same amount of supplemental Lys was greater when overall protein in the diets was lower. This suggests that dietary protein content modifies the effects of free Lys supplementation and low protein intake may exacerbate the effects of Lys supplementation. The upper tolerant levels of Lys supplementation may be dependent on dietary protein intake and determined by the ratio of supplemental free Lys to the amounts of protein consumed. This is supported by the strong inverse correlation among the ratios of supplemental Lys to dietary protein content and protein quality of the diets and BWG.

The present study showed that supplemental free Lys led to higher serum concentrations of Lys compared to similar levels of protein-bound Lys. This suggests higher intestinal absorption rates of free Lys as serum free AA concentration is indicative of absorption and availability^31^. Free Lys was absorbed more rapidly than protein-bound Lys in pigs^32^. Similar observations have been reported in studies on other AA in other species^33–35^. The efficiency of AA absorption was improved when both protein-bound and free AA were included in the diet^31^. The form in which AA is consumed affects its immediate metabolic fate and retention by the body in humans^36^. Protein-bound AA are believed to have much less effect on their serum levels as protein intake stimulates protein synthesis^36–38^.

Supplemental Lys reduced serum ornithine levels but had no significant effect on serum Arg and citrulline levels. This suggests that arginase, responsible for the conversion of Arg to ornithine, might have been inhibited by supplemental Lys. This is consistent with our proteomic analysis results showing decreased arginase-1 protein abundance in the livers of rats fed a 7% casein diet with 1.5% supplemental Lys compared to the Control (Supplementary Fig. 1 and Table 3). Furthermore, Lys is a competitive inhibitor of arginase^39^, and therefore addition of 3% Arg along with 6% Lys eliminated the inhibitory action of supplemental Lys on serum ornithine concentrations. Meanwhile, addition of 3% Arg also attenuated the increase in serum Lys concentrations in response to Lys supplementation. Similar effects of Arg on serum Lys have been observed in other species^40–42^. This effect may be explained by decreased absorption of Lys as a consequence of competitive inhibition of membrane transport between Lys and Arg in the intestinal mucosa^43^ and renal tubule^44,45^.

Effects of Lys supplements on blood levels of Arg and Arg metabolites may vary depending on the paths and doses of Lys administration as well as levels of dietary protein intake. Intravenous infusion of Lys monohydrochloride in human subjects increased plasma Arg, ornithine, blood ammonia, and urinary homocitrulline levels, which appears to reflect inhibited arginase activity and conversion of ornithine to citrulline via ornithine transcarbamylase. There was little change in plasma urea and citrulline^46^. Our study showed that serum urea but not citrulline and ammonia levels were increased in rats fed the 7% casein diets by supplemented Lys, but no changes were observed in rats fed 20% casein diets.

Supplemental Lys at ≥ 3% in 7% casein or at 6% in 20% casein diets markedly reduced BWG and food intake as reported previously^28^. Supplemental Lys at ≥ 1.5% markedly lowered serum branched chain amino acids (BCAA) (Ile, Leu, Val) and 5 non-EAA (Ala, Asn, Gly, Ser and Tyr) concentrations in the rats fed the 7% casein diet, and Asn and Thr in rats fed the 20% casein diet compared to the Controls. Supplemental Lys at ≥ 3% in 7% casein or 6% in 20% casein diets markedly increased serum His and Met levels. Addition of Arg fully or partially reversed the effects of supplemental Lys on food intake, BWG, serum Lys, Tyr, His and Met, indicating antagonism between Lys and Arg. Arg supplementation also partially attenuated the growth retardation induced by 4.3% Lys supplementation in rats^47^.

In addition to Lys antagonism on Arg, some effects of Lys supplementation may be attributed to other mechanisms such as inhibition of AA absorption and metabolism, resulting in AA imbalance. In pigs, feeding diets with 3.45% supplemental Lys in the background of marginal Arg lowered both BWG and feed intake, and increased free Lys and α-AAA levels in plasma, liver, kidney, and muscle tissues compared to the basal diet. However, the tissue levels of arginase and ornithine transcarbamoylase were unchanged by supplemental Lys^48^. Increased dietary Lys levels reduced two of the three plasma BCAA (Ile, and Val) and Thr concentrations in growing barrows^49^, or plasma Thr, Tyr and some non-EAA in finishing pigs^50^. The mRNA abundances of cationic AA transporters in the small intestine of pigs were shown to be lowered by high dietary Lys levels^51^, which may play a role in mediating the reduction of neutral AA such as Ala, Gly, Leu, and Ile through suppression of intestinal absorption.

Dietary amino acid imbalance, particularly deficiencies in essential amino acids, can trigger cellular stress response pathways such as the integrated stress response and the amino acid response^52^. Activation of these pathways leads to a reduction in protein synthesis and cell growth as a means of conserving energy and resources during periods of amino acid scarcity. The general control nonderepressible-2 kinase is a sensor for amino acid imbalance in activating the integrated stress response leading to inhibition of protein synthesis and extended longevity^53–55^. Target of Rapamycin (TOR) kinase, a central regulator of cell growth and metabolism, is tightly regulated by amino acid levels^52^. When amino acid imbalance occurs, TOR kinase activity can be inhibited, which can further induce autophagy, a cellular process that helps remove damaged cellular components, contributing to cellular maintenance and overall health. Furthermore, reduced TOR activity has been associated with increased lifespan in various organisms^56,57^, suggesting a potential link between amino acid imbalance-induced TOR inhibition and longevity.

α-AAA, structurally similar to glutamine, is an intermediate of Lys metabolism, and competitively inhibits the activity of glutamine synthetase (Glns), γ-glutamylcysteine synthetase and the high–affinity glutamate/aspartate transporter^58^. Our previous study showed that dietary Lys supplementation reduced serum glutamine levels and Glns abundance in skeletal muscle of rats fed a 7% casein diet^28^. The present study showed that supplementation of ≥ 3% Lys in 7% casein or 6% Lys in 20% casein markedly increased serum α-AAA. This suggests that supplemental Lys may have a dual effect on Glns, reducing its protein abundance along with inhibition of its activity through increased α-AAA. α-AAA is an emerging predictor of the risk for development of diabetes in both normoglycemic and high CVD risk individuals^59,60^. The baseline α-AAA circulating level was associated with future risk of type-2 diabetes and was increased up to 12 years before the onset of diabetes and the development of other known risk markers. α-AAA was also associated with obesity and metabolic syndrome^60–62^. α-AAA may modulate insulin secretion and plasma glucose levels^60,63^, impair insulin signalling, result in abnormal gluconeogenesis^61^ and reduce high-density lipoprotein cholesterol level^62^.

Dietary supplementation with Lys significantly lowered serum carnitine concentrations and this effect was dependent on the protein content of the diets. All levels of Lys supplementation in 7% casein lowered serum carnitine levels, however, only the highest level of Lys (6%) added in the 20% casein diet had this effect. Excess dietary Lys was shown to reduce plasma carnitine concentrations, but increase the concentrations of free trimethyllysine (TML), a carnitine precursor, in skeletal muscle and plasma of rats^64^. Further research in pigs suggested that the potential mechanism underlying this effect of Lys may involve downregulation of the mRNA expression of muscle TML dioxygenase, a rate-limiting enzyme responsible for conversion of TML to γ-butyrobetaine (BB). Impaired conversion of TML to BB resulted in increased TML concentrations and decreased BB in muscles, reducing serum carnitine levels as BB is the precursor of carnitine biosynthesis^65^.

In conclusion, this study showed that addition of Lys in Lys-sufficient diets reduced the limiting EAA (Met + Cys) scores and protein quality in a dose-dependent manner when dietary protein was adequate (20% casein) or low (7% casein). The percentage of reduction by the same amount of Lys supplementation was greater when protein was low. The ratios of supplemental free Lys to dietary protein content were inversely correlated with protein quality of diets, BWG and utilization of nutrients. Dietary protein-bound and free Lys and Arg differentially affect blood circulating Lys and Arg levels. Compared with protein-bound Lys, similar levels of supplemental free Lys markedly increased serum Lys. Moreover, circulating amino acid profiles and many of their metabolites were significantly altered. Overall, these results suggest that Lys supplementation adversely affected protein quality of diets and resulted in growth retardation, and low protein intakes exaggerate the adverse effects from Lys supplementation. Further investigation is warranted to explore the potential long-term impact of Lys and Arg supplementation on the regulation of circulating sulfur amino acid concentrations and its relationship to longevity.

## Materials and methods

### Animals and diets

The animal experimental protocol (No. 2014-018) was approved by the Health Canada Animal Care Committee, and all animal handling and care followed the guidelines of Canadian Council for Animal Care. The reporting in this paper followed the recommendations in the ARRIVE guidelines^66^. Male SAS Sprague Dawley rats (Strain Code: 400) at 9 weeks of age from Charles River (St. Constant, Quebec, Canada) were housed individually and placed on a 12:12 h light:dark cycle, with free access to food and water. After acclimation for 1 week on a 20% casein diet, the rats were assigned to 10 groups (8 rats/group) based on their body weights to ensure that the mean initial body weights were similar among groups. The sample size calculation was conducted based on BWG with a 25% standard deviation to detect a 35% reduction. The power of the experiment was set to 80%. A minimum sample size of 8 was considered necessary. The study is a two-way randomization trial with 80 rats. The grouped rats were randomly assigned to one of the 10 experimental diets, which contained either 7% or 20% casein and were supplemented with 0 (Control), 1.5%, 3%, 6% of Lys or 6% Lys + 3% Arg for 1 week. The rats in cages were randomly located on the rack. The diets were formulated following the AIN-93G recommendations for rodents^67^, and changes in casein and Lys or Arg contents were at the expense of cornstarch or sucrose, respectively. The digestible energy content of the diets containing the same levels of casein (7% or 20%) were equivalent. Titanium dioxide (TiO_2_) at a level of 0.3% was added to all diets as an indigestible marker (Supplementary Table 1). Food consumption and body weight were measured at the end of the study. On the day before necropsy, all rats were moved to metabolic cages with food for collection of urine and feces. After a 4-h fast, rats were euthanized by exsanguination from the abdominal aorta while under 3% isoflurane anesthesia through inhalation as recommended^68^. Blood and liver were collected for isolation of serum and proteomics analysis.

### Measurement of total nitrogen and TiO_2_ content in diets and feces

Diets and feces were freeze-dried and ground. Total nitrogen (TN) content was determined by Combustion Analysis using the Association of Official Agricultural Chemists Official Method 990.03, 2006. Crude protein was calculated using a conversion factor of 6.25 from TN. TiO_2_ content was measured using the procedure as described by Myers et al.^67^.

### Fecal true protein digestibility

The fecal true protein digestibility (TPD) was calculated using the TN-to-TiO_2_ ratio in the diets and feces according to the following equation^69^:

$$ {\text{Fecal}}\,{\text{true}}\,{\text{protein}}\,{\text{digestibility}}\,\left( \% \right)\,\, = \,100\, \times \,\left[ {\left( {TN/TiO_{2} } \right)_{diet} - \left( {\left( {TN/TiO_{2} } \right)_{feces} - F_{k} } \right)} \right]/\left( {TN/TiO_{2} } \right)_{diet} . $$

The TN-to-TiO_2_ ratio in the feces of the rats on a protein-free diet or endogenous fecal nitrogen excretion (F_k_) from a separate study with the same design (Supplementary Table 1) was used in the calculations.

### Determination of amino acids in diet and serum samples

Serum samples were deproteinized using sulfosalicylic acid solution, and the free amino acids and some of their metabolites were analysed using automated cation-exchange column liquid chromatography with post column ninhydrin reaction and dual wavelength detection in a Beckman high standard amino acid analyser. Amino acids in the diets were determined using the Association of Official Agricultural Chemists Official Method 982. 30 E (a,b) chp. 45.3.05, 2006. Serum carnitine was measured using the carnitine assay kit (Abcam Inc., Toronto, Canada). Serum ammonia levels were determined using the ammonia assay kit (Sigma-Aldrich, Oakville, Canada).

### EAA scores and protein quality in diets

The EAA scores in diets were the lowest ratios of the EAA content (mg/g protein) to their requirements^30^ in rats. The protein quality of the diets was determined using Protein Digestibility-Corrected Amino Acid Score (PDCAAS). PDCAAS = the limiting EAA score × TPD.

### Statistical analysis

Data are presented as mean ± SEM. All data were evaluated for equality of variance prior to statistical analysis. Variables with skewed distribution were logarithmically transformed. The effects of different levels of dietary protein and supplemental Lys as well as their interaction were analyzed by two-way Analysis of Variance. Differences between individual means were determined by Tukey’s honestly significant difference *post-hoc* test. A probability of p < 0.05 was considered to be significant. The correlation between the ratios of supplemental Lys to protein content and PDCAAS was assessed using a linear regression model. The correlation between the ratios of supplemental Lys to protein content and BWG was analysed using segmented linear regression modelling from the ‘chngpt’ R-package (4.03). Its breakpoint and 95% confidence intervals were estimated using a grid search method and bootstrap approach, respectively^70–72^. All other data were analyzed using STATISTICA version 13 (StatSoft, Inc., Tulsa, OK, USA).

### Ethics approval

The animal experimental protocol (ACC#2014-018) was approved by the Health Canada-Ottawa Animal Care Committee, and all animal handling and care followed the guidelines of the Canadian Council for Animal care.

### Supplementary Information

Supplementary Figure 1.

Supplementary Table 1.

Supplementary Table 2.

Supplementary Table 3.

## Data Availability

The datasets generated during and/or analysed during the current study are available from the corresponding author on reasonable request.

## Abbreviations

α-AAA: α-Aminoadipic acid
AA: Amino acid
Arg: l-arginine
BB: γ-Butyrobetaine
BCAA: Branched chain amino acids
BWG: Body weight gain
EAA: Essential amino acid
Glns: Glutamine synthetase
Lys: l-lysine
PDCAAS: Protein digestibility-corrected amino acid score
TML: Trimethyllysine
TN: Total nitrogen
TPD: True protein digestibility
