# Supplementary material for: l-Lysine supplementation affects dietary protein quality and growth and serum amino acid concentrations in rats
Source: Sci Rep. 2023 Nov 15;13:19943. doi: 10.1038/s41598-023-47321-3 (PMC10651908; doi:10.1038/s41598-023-47321-3)
Supplement: Supplementary file 1 — Supplementary Figure 1. [file 41598_2023_47321_MOESM1_ESM.pptx]

## Slide 1
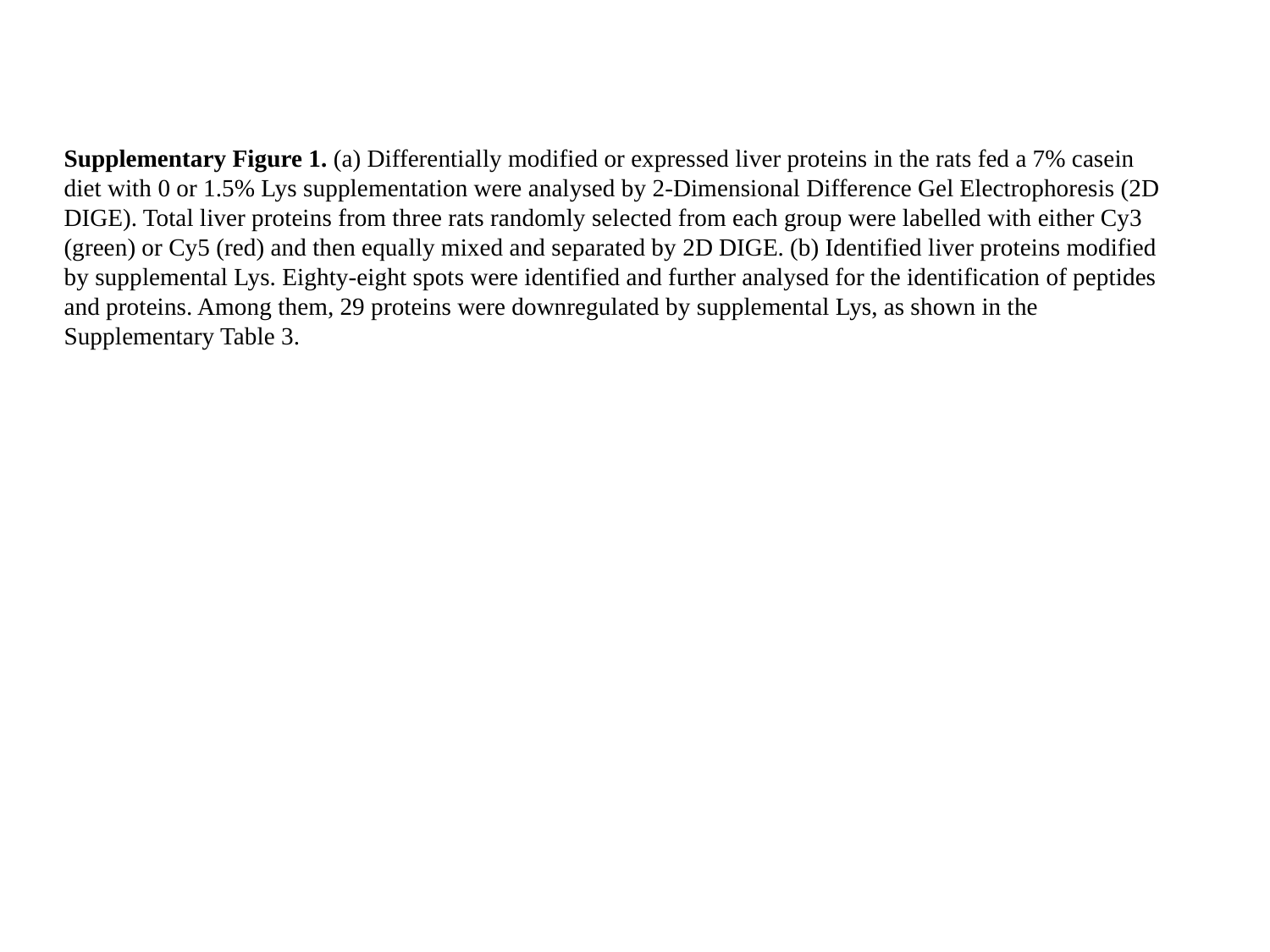

Supplementary Figure 1. (a) Differentially modified or expressed liver proteins in the rats fed a 7% casein diet with 0 or 1.5% Lys supplementation were analysed by 2-Dimensional Difference Gel Electrophoresis (2D DIGE). Total liver proteins from three rats randomly selected from each group were labelled with either Cy3 (green) or Cy5 (red) and then equally mixed and separated by 2D DIGE. (b) Identified liver proteins modified by supplemental Lys. Eighty-eight spots were identified and further analysed for the identification of peptides and proteins. Among them, 29 proteins were downregulated by supplemental Lys, as shown in the Supplementary Table 3.

## Slide 2
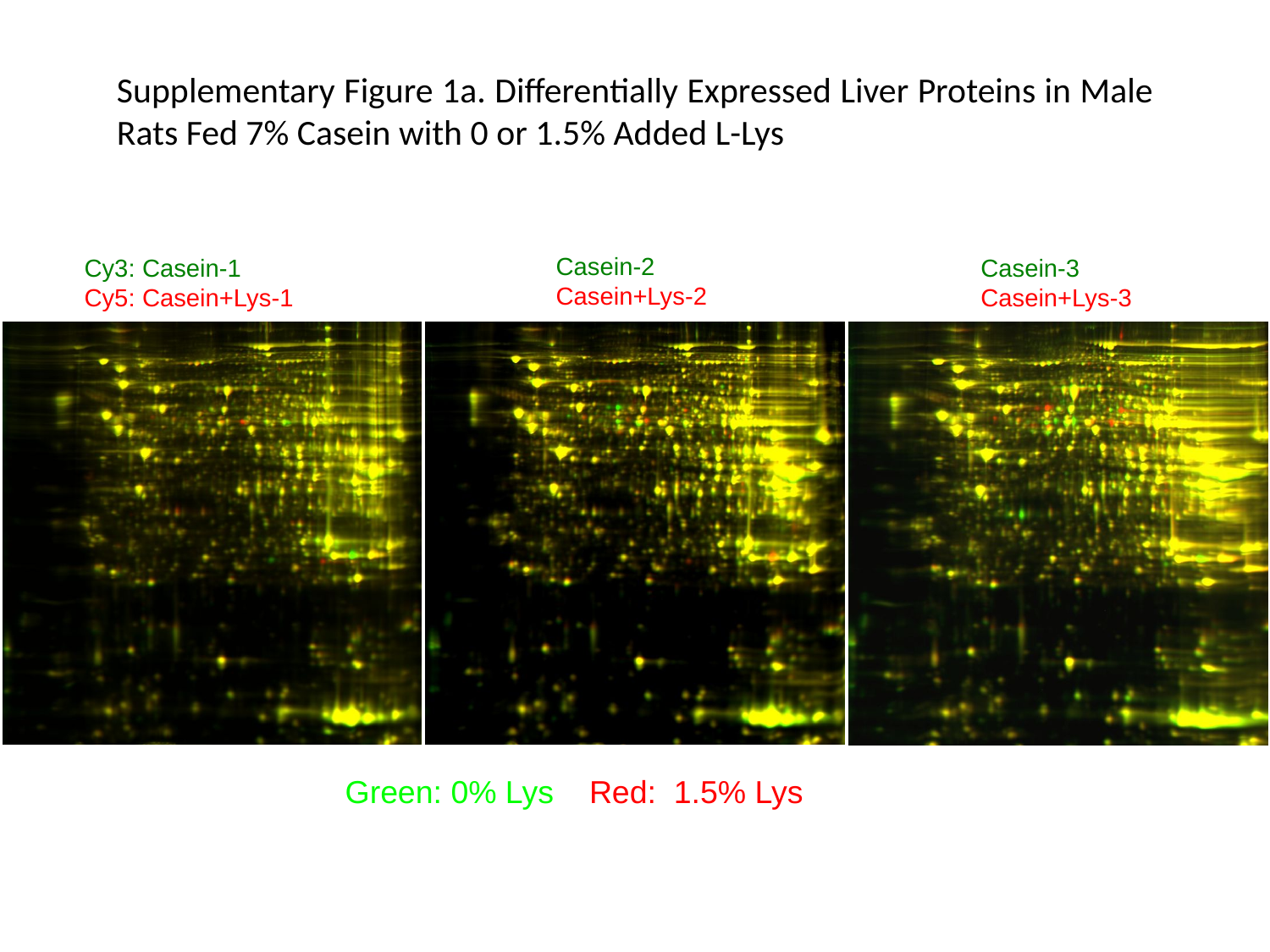

Supplementary Figure 1a. Differentially Expressed Liver Proteins in Male Rats Fed 7% Casein with 0 or 1.5% Added L-Lys
Casein-2
Casein+Lys-2
Cy3: Casein-1
Cy5: Casein+Lys-1
Casein-3
Casein+Lys-3
Green: 0% Lys Red: 1.5% Lys

## Slide 3
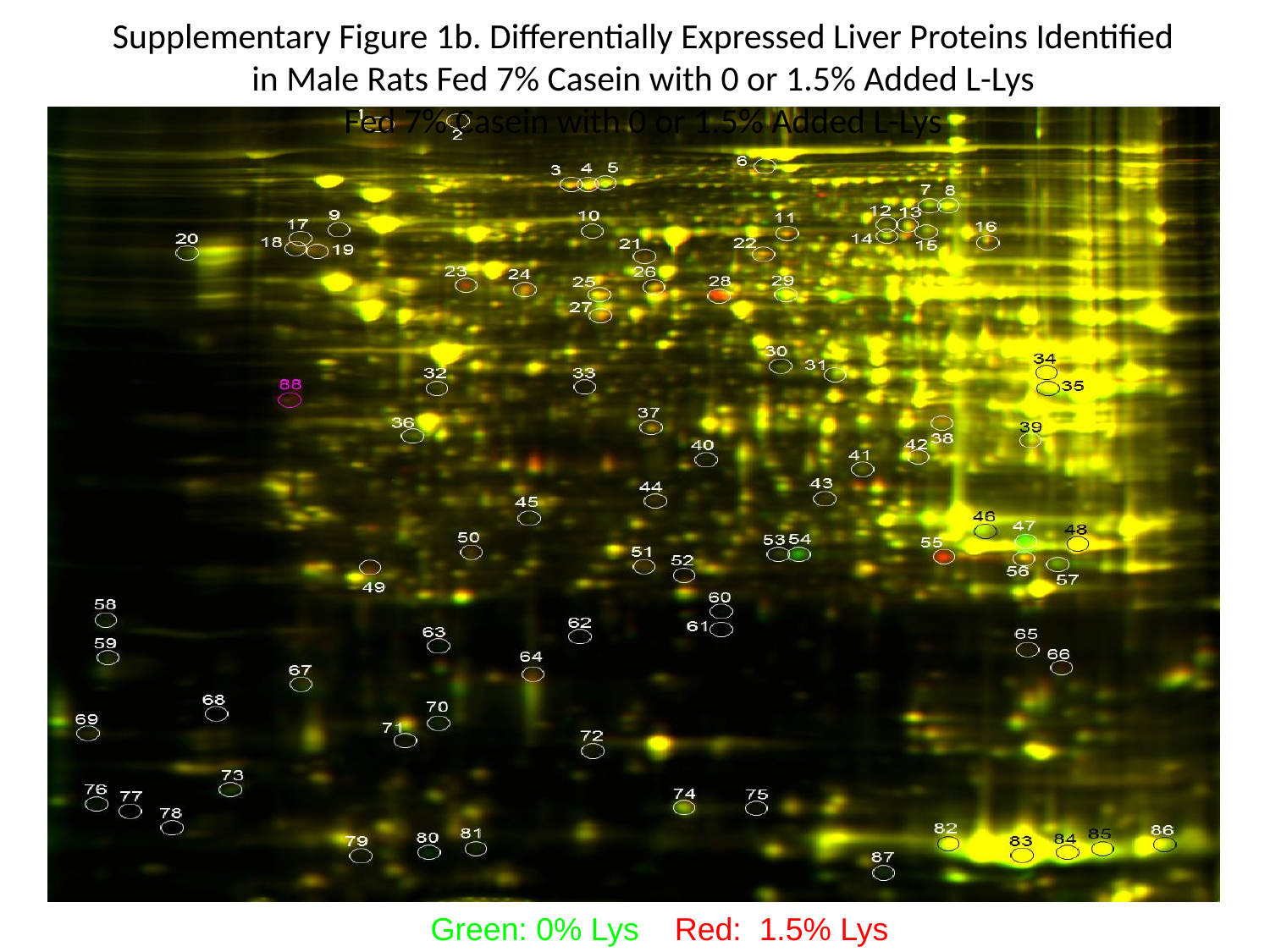

Supplementary Figure 1b. Differentially Expressed Liver Proteins Identified in Male Rats Fed 7% Casein with 0 or 1.5% Added L-Lys
Fed 7% Casein with 0 or 1.5% Added L-Lys
Green: 0% Lys Red: 1.5% Lys
3
