# Supplementary material for: l-Lysine supplementation affects dietary protein quality and growth and serum amino acid concentrations in rats
Source: Sci Rep. 2023 Nov 15;13:19943. doi: 10.1038/s41598-023-47321-3 (PMC10651908; doi:10.1038/s41598-023-47321-3)
Supplement: Supplementary file 2 — Supplementary Table 1. [file 41598_2023_47321_MOESM2_ESM.docx]

Supplementary Table 1. Composition of experimental diets
