# Supplementary material for: l-Lysine supplementation affects dietary protein quality and growth and serum amino acid concentrations in rats
Source: Sci Rep. 2023 Nov 15;13:19943. doi: 10.1038/s41598-023-47321-3 (PMC10651908; doi:10.1038/s41598-023-47321-3)
Supplement: Supplementary file 3 — Supplementary Table 2. [file 41598_2023_47321_MOESM3_ESM.docx]

Supplementary Table 2. Amino acid, total crude protein content and ratios of supplemental lysine to crude protein in diets *CP: Crude protein = %N × 6.25; **Ratios of supplemental Lys to CP.
